# Major ozonated autohemotherapy promoted functional recovery following spinal cord injury in adult rats via the inhibition of oxidative stress and inflammation

**DOI:** 10.1515/biol-2022-1004

**Published:** 2024-12-31

**Authors:** Liwei Xia, Yongming Sun, Yue Zhou, Qian Yang, Jianhan Huang, Dong Liu

**Affiliations:** Department of Orthopaedics, The Second Affiliated Hospital of Soochow University, Suzhou, Jiangsu, 215004, China; Department of Clinical Medicine, Suzhou Medical College of Soochow University, Suzhou, 215000, China; Department of Orthopaedics, Guangxi Zhuang Autonomous Region Jiangbin Hospital, Nanning, 532001, China

**Keywords:** SCI, major ozonated autohemotherapy, inflammation, superoxide dismutase

## Abstract

This study sought to explore the value of major ozonated autohemotherapy (MOA) as a treatment for spinal cord injury (SCI) in a rat model system. In total, 54 female Sprague-Dawley rats were randomized into sham-operated, SCI model, and MOA treatment groups. We found that relative to the SCI model group, rats that underwent MOA treatment exhibited improved locomotor scores on days 14, 21, and 28 after injury (*p* < 0.05) together with reduced residual urine on days 5, 7, 14, and 21 after injury (*p* < 0.05). MOA treatment also lowered proinflammatory TNF-α, IL-1α, and C1q levels on day 3 post-injury (*p* < 0.05), decreased malondialdehyde levels, and enhanced superoxide dismutase activity (*p* < 0.001). Activated astrocytes in MOA-treated rats exhibited larger soma and higher levels of extracellular matrix secretion, whereas reactive microglia in the MOA group presented with a ramified morphology in contrast to the amoeboid morphology exhibited by these cells in SCI model rats. MOA offers potential value as a means of protecting spinal cord integrity, potentially through anti-inflammatory, antioxidant, and regulatory effects that shape the polarization of astrocytes and microglia.

## 1 Introduction

Spinal cord injury (SCI) represents an intractable medical issue that remains difficult to effectively manage. While countless studies have explored the pathogenesis of SCI and associated molecular mechanisms, treatment options for SCI patients remain limited and there is no clinical consensus regarding their application [1]. Following initial injury, inflammatory and oxidative stress in response to spinal trauma can induce the apoptotic or necrotic death of neurons while driving the formation of a spinal cord cavity, ultimately compromising spinal cord functionality [2,3]. In this pathological context, microglia can differentiate into deleterious M1-type microglia that secrete inflammatory factors including TNF-α, IL-1β, proteolytic enzymes, and nitric oxide synthase, ultimately inducing unrestrained inflammation that aggravates neuronal death and associated tissue damage [4,5]. Efforts to prevent severe inflammatory responses and oxidative stress induction following SCI have thus emerged as the preferred approach to treating SCI cases.

Medical ozone has been reported to be a safe and effective non-pharmacological treatment option with antioxidant, anti-inflammatory, and antibacterial properties [6,7,8,9]. Major ozonated autohemotherapy (MOA) is a treatment approach wherein autologous blood samples are mixed with medical ozone (containing O_2_ and O_3_) followed by gradual infusion back into the host. MOA has been used to treat a range of conditions including osteonecrosis, acute cerebral infarction, lumbar disc herniation, vascular, and immunological diseases [8,9,10,11,12].

MOA therapy can effectively alleviate oxidative stress in emergency settings, and can also reportedly protect against cytokine storm induction when treating COVID-19, thereby providing a promising means of preventing hospitalized COVID-19 patient mortality [13,14]. Ozone can also alleviate neuronal damage following acute cerebral infarction through oxygen free radical scavenging and the activation of antioxidant enzymes. Yu et al. [15] found that a patient who was paraplegic following a spontaneous spinal epidural hematoma exhibited significant lower extremity muscle contractions 5 days following MOA treatment, ultimately achieving 3/5 strength in their lower extremities after two rounds of treatment. Ameli et al. [16] suggested that ozone therapy may be an effective therapeutic option for patients with multiple sclerosis owing to its ability to alleviate oxidative stress and inflammation while increasing blood oxygen concentrations. Tural Emon et al. [17] further observed accelerated SCI healing in rodents that were subjected to ozone therapy, suggesting that it may represent a valuable complementary means of treating SCI patients. However, the ability of MOA to promote the repair of spinal cord damage through the suppression of inflammation and oxidative stress remains to be studied and documented in detail.

In the present study, MOA was found to effectively contribute to motor function recovery in adult rats following SCI. At the mechanistic level, MOA suppressed inflammation, prevented the M1 differentiation of microglia, and effectively promoted the growth of neuronal axons and dendrites. As such, MOA holds promise as a novel treatment for SCI.

## 2 Materials and methods

### 2.1 Establishment of the cellular OGD model

Primary spinal cord neurons were isolated and cultured. Female SD rats were obtained from the Animal Center of the University of Guangxi Medical University. Fourteen days after the start of pregnancy, the rats were anesthetized in a sterile environment. The spinal cord of the embryos was harvested, digested with 0.125% trypsin at 37°C for 15 min, and then digested with an equal volume of an inoculant solution (Dulbecco’s modified eagle medium (DMEM) containing 10% fetal bovine serum and 10% equine serum; Sigma-Aldrich, St Louis, MO, USA). The cells were then centrifuged at 1,000 rpm for 5 min (centrifugation radius 10 cm) and the middle layer containing single cells was collected. These cells were inoculated into polylysine-coated sterile six-well plates (2 ml/well) at a density of 1 × 10^5^ cells/ml. When the purity of the neuron cells was greater than 90%, the culture supernatant was removed and the cells were rinsed three times with normal saline, then added 5% CO_2_–95% N_2_ sugar-free Earle’s balanced salt solution. Lastly, the cells were placed in an anoxic incubator at 37°C (5% CO_2_–95% N_2_) and incubated for 2 h, after which they were returned to the neuron culture medium and grown at 37°C with 5% CO_2_–95% air. The cells were then divided into the control, OGD, and OGD + MOA groups.


**Ethical approval:** The research related to animal use has been complied with all the relevant national regulations and institutional policies for the care and use of animals and has been approved by the Ethics Committee of the Second Affiliated Hospital of Soochow University.

### 2.2 Animals model establishment

Adult female Sprague-Dawley rats (250–270 g) were obtained from the Animal Center of the University of Guangxi Medical University in Nanning, China. An experimental model of SCI was established using Allen’s method. Briefly, rats were intraperitoneally injected with 1% pentobarbital sodium (60 mg/kg) and fixed in the prone position, after which a longitudinal incision was used to expose the T10 spinous process, followed by the excision of the T10 lamina and the exposure of the spinal cord. The exposed spinal cord (approximately 4 mm × 10 mm) was then impacted using a multi-center animal spinal cord injury study (MASCIS) impounder to hit the exposed spinal cord (10 g × 6 cm). Successful SCI modeling was indicated by rapid dural swelling, bruising, lower extremity convulsions, and a tail-wagging reflex. After surgery, rats received intramuscular injections of penicillin (0.8 mg/g) for 3 days. The bladder of each rat was emptied twice per day until autonomous urination had resumed. Rats in the sham control group underwent T9-10 laminectomy without SCI. The procedures were approved by the Ethics Committee of the Second Affiliated Hospital of Soochow University.

### 2.3 Ozonated autohemotherapy

Pure oxygen was used to produce ozone with a medical ozone generator (Kasener-Praxisbedarf Gmbh, Rastatt, Germany). Samples of blood (1 ml) were collected from the tail vein and immediately mixed with the produced ozone for 5 min, followed by the re-infusion of the ozonated blood through the tail vein. MOA was performed once per day on 3 consecutive days.

### 2.4 Behavioral testing

Two investigators independently performed rat behavioral assessments with the BBB Locomotor Rating Scale prior to surgery and on days 1, 3, 7, 14, 21, and 28 post-SCI. Each rat was analyzed three times, with the average value being reported.

### 2.5 Bladder function analyses

The bladders of injured rats were manually emptied twice per day after injury, with the obtained urine being collected as a measure of bladder function. Residual urine volumes were measured for each rat on days 1, 2, 3, 5, 6, 14, and 21 post-injury.

### 2.6 MOA treatment of RAW264.7 cells

The mouse macrophage cell line RAW264.7 was purchased from Wuhan Procell Life Science & Technology Co., Ltd. The cells (2.0 × 10^4^/well) were plated in 24-well plates in DMEM containing 10% fetal bovine serum, divided into the control, lipopolysaccharide (LPS) + phosphate buffered saline (PBS), and LPS + MOA groups, and treated with anti-CD86 (ab239075, Abcam, UK) and anti-CD206 (anti-mannoreceptor antibody, ab125028, Abcam). Macrophage polarization was observed by confocal laser scanning microscopy (CLSM, LSM800 with Airyscan, Zeiss, Germany).

### 2.7 Histological staining

Sections of spinal cord tissue from the 10th thoracic segment were collected, fixed using paraformaldehyde, paraffin-embedded, and cut into serial 5 µm sections followed by hematoxylin and eosin (H&E) staining. Sections were then imaged using a light microscope (XSP-C204, CIC).

### 2.8 NeuN/4′,6-diamidino-2-phenylindole (DAPI) staining

After OGD treatment, NSC cells were fixed with 4% paraformaldehyde solution for 30 min and then permeabilized with 0.1% Triton X-100 for 10 min. FITC-phalloidin (Yeasen, Shanghai, China) and βIII Tubulin (Yeasen) were used to stain actin in the cytoskeleton, and the nuclei were counterstained with DAPI for 5 min at 37°C. The cells were examined and imaged using a laser-scanning confocal microscope. Cell numbers and morphology were analyzed by ImageJ software.

### 2.9 Western immunoblotting

Samples of spinal cord tissue (1 cm long) were isolated and lysed on ice in a lysis buffer supplemented with protease and phosphatase inhibitors. A bicinchoninic acid kit was used to assess protein concentrations, after which proteins were separated via 10 or 12.5% SDS-PAGE and transferred onto polyvinylidene fluoride membranes. Blots were then blocked for 1 h using 5% BSA followed by incubation overnight with antibodies specific for TNF-α (1:500; Novusbio), IL-1α (1:1,000; Novusbio), C1q (1:1,000; CST), anti-HO-1 (1:10,000; Abcam), anti-Nrf2 (1:500; Thermo), or SOD (1:1,000; CST) at 4°C. After probing for 1 h at room temperature using HRP-conjugated secondary antibodies, ImageJ (v1.48) was used to analyze densitometric values for detected protein bands, with β-actin serving as a loading control.

### 2.10 PCR

TRIzol (Thermo Fisher Scientific, MA, USA) was used to extract RNA from spinal cord tissue samples based on provided directions. The QuantiNova SYBR Green PCR Kit (QIAGEN) and an ABI 7500 platform (Applied Biosystems, USA) were used for qPCR analyses, with β-actin as a normalization control. The reaction conditions were: 95°C for 10 min, 95°C for 10 s, 60°C for 20 s, 72°C for 30 s, 40 cycles. The 2^–ΔΔCt^ method was used to compare relative gene expression levels across treatment groups. Primer sequences used for this study are listed below (Table 1).

**Table 1:** Primer sequences of TNF-α, Rat-IL-1α, Rat-IL-1β, Rat-IL6, HO-1, Nrf2, Rat-C1q, and Rat-β-actin

Gene	Forward	Reverse
TNF-α	ATGGGCTCCCTCTCATCAGT	TGGTGGTTTGCTACGACGTG
Rat-IL-1α	CGCTTGAGTCGGCAAAGAAATC	AGAGACAGATGGTCAATGGCAG
Rat-IL-1β	GATGATGACGACCTGCTAGTGTGT	TTGGCTTATGTTCTGTCCATTGAG
Rat-IL6	CTTCCAGCCAGTTGCCTTCTT	GGTCTGTTGTGGGTGGTATCCT
HO-1	TCAAGGCCTCAGACAAATCC	ACAACCAGTGAGTGGAGCCT
Nrf2	TGCCTCCAAAGGATGTCAAT	CCTCTGCTGCAAGTAGCCTC
Rat-C1q	GACCACGGAGGCAGGAACATC	AATTCCTGCAACCCCGTCCT
Rat-β-actin	GGTGGGGCGCCCCAGGCACCA	GCTCCTTAATGTCACGCACGA

### 2.11 Malondialdehyde (MDA) and superoxide dismutase (SOD) activity assays

Levels of MDA (nmol/l) and SOD activity (U/mg) in spinal cord tissue were assessed with commercial kits (Nanjing Jiancheng Biotech, Nanjing, China). These spinal cord samples were collected 3 days following SCI, suspended in PBS (pH 7.4), homogenized completely with a homogenizer, and centrifuged at 3,000 rpm for 20 min, and supernatants were collected for analysis.

### 2.12 Immunofluorescent and immunohistochemical staining

Changes in astrocyte and microglial morphology were assessed on day 3 post-SCI via GFPA and Iba immunostaining. Briefly, 4-µm tissue sections were rinsed with PBS, blocked with 3% BSA, and treated with an autofluorescence quenching kit (G1221, ServieBio). These sections were then incubated overnight with primary rabbit anti-GFAP (Abcam, ab33922) or rabbit anti-Iba1 antibody (Abcam, ab178847) for immunofluorescent or immunohistochemical staining, respectively. Sections were then proved with secondary antibody goat Anti-Rabbit IgG (HRP) (1:500, GB23303, ServiceBio) for 50 min at room temperature, followed by visualization with a fluorescence microscope (Nikon Eclipse C1, Japan). Changes in NSC morphology were assessed via GFPA and Iba immunostaining.

### 2.13 Statistical analysis

In this experiment, the data were statistically analyzed by Graphpad Prism, and the mean ± standard deviation was used to represent the data. The *t*-test was used for the comparison between the two groups, and the data conforming to the normal distribution were used. The non-parametric Wilcoxon rank-sum test was used for the non-parametric data that did not conform to the normal distribution. *p* < 0.05, which was considered statistically significant. In this experiment, the western blot band plot was processed using ImageJ image analysis software.

## 3 Results

### 3.1 MOA treatment restores locomotor function and bladder function following SCI

**Figure 1:**
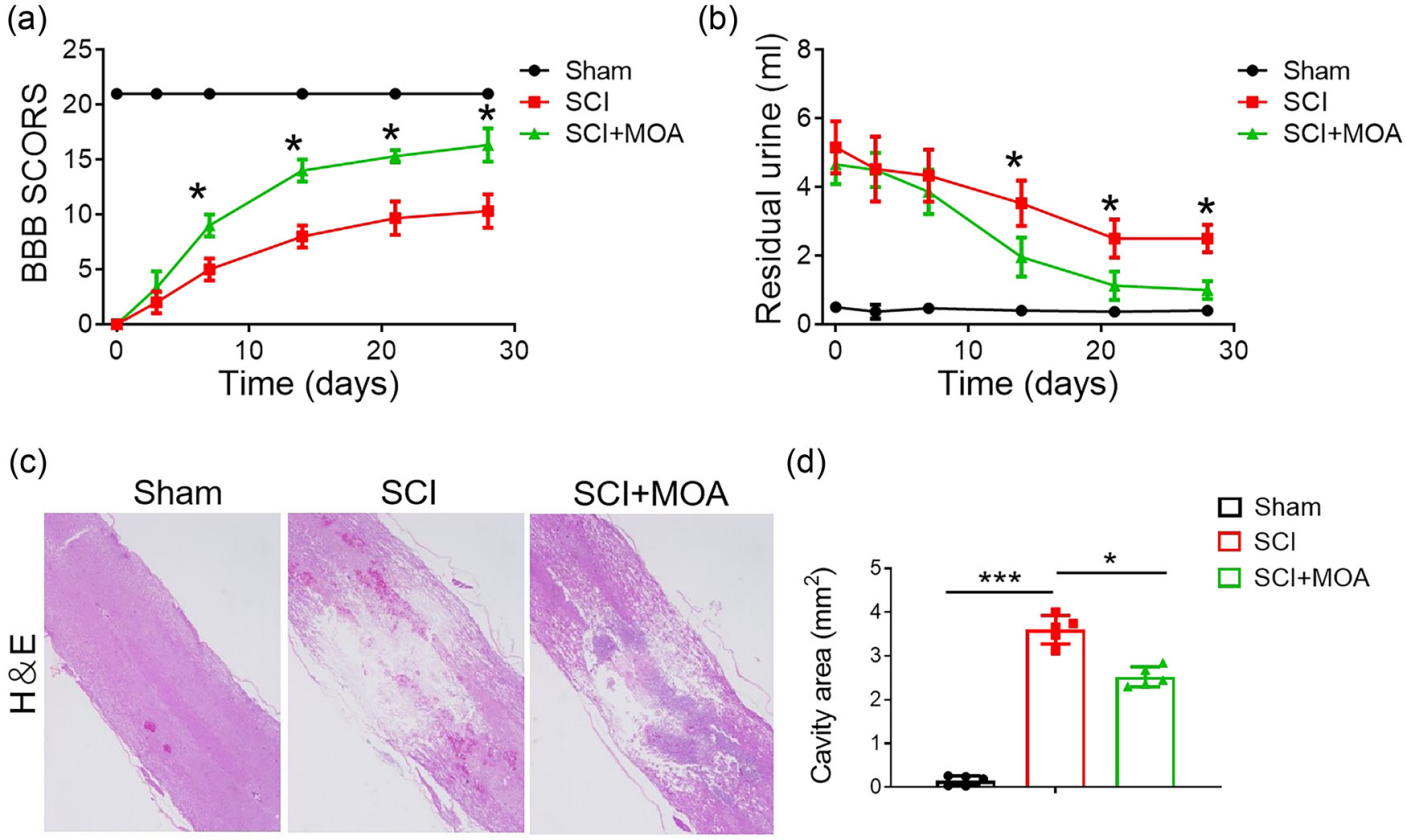
(a) BBB scores of each group, *p < 0.05 versus the SCI group, *n* = 8. (b) Residual urine volumes in each group, *p < 0.05 versus the SCI group, *n* = 8. (c) HE staining of spinal cord sections in the sham, SCI, and MOA groups. (d) Cavity area of HE staining in each group, *p < 0.05 versus the SCI group.

Locomotor recovery after SCI was initially assessed in experimental rats using BBB scores. BBB scores for rats in the SCI group were very low after injury with no significant differences between the MOA and SCI groups for the first week post-injury. However, these scores were significantly higher for rats in the MOA treatment group on days 7, 14, 21, and 28 post-injury (9.4 ± 1.7, 13.8 ± 1.5, 14.1 ± 1.6, 14.9 ± 1.9) relative to the SCI group (5.6 ± 1.0, 10.0 ± 1.6, 11.1 ± 1.9, 11.8 ± 1.3) (Figure 1a). This suggests that MOA treatment significantly enhanced locomotor recovery in these rats (*p* < 0.05, *n* = 8/group).

The recovery of bladder function in all experimental groups was assessed based on residual urine volume. At 1 day post-injury, the residual urine volume in the SCI and MOA groups was 5 ml, with this value decreasing over time, particularly in the MOA group (Figure 1b). While no significant differences in residual urine volume were evident between the MOA and SCI groups at early time points, on days 5, 7, 14, and 21 post-injury this volume was significantly larger in the SCI group relative to the MOA group (*p* < 0.05).

### 3.2 MOA protects against necrotic tissue damage in the spinal cord

On day 3 post-injury, histologic analyses of rat spinal cord tissue samples were performed. Morphological analyses revealed extensive edema, hemorrhage, necrosis, and cavity formation in the SCI model group, whereas these necrotic phenotypes were markedly blunted in the MOA treatment group, suggesting that MOA can protect against severe damage following SCI (Figure 1c and d).

### 3.3 MOA suppresses proinflammatory cytokine expression

**Figure 2:**
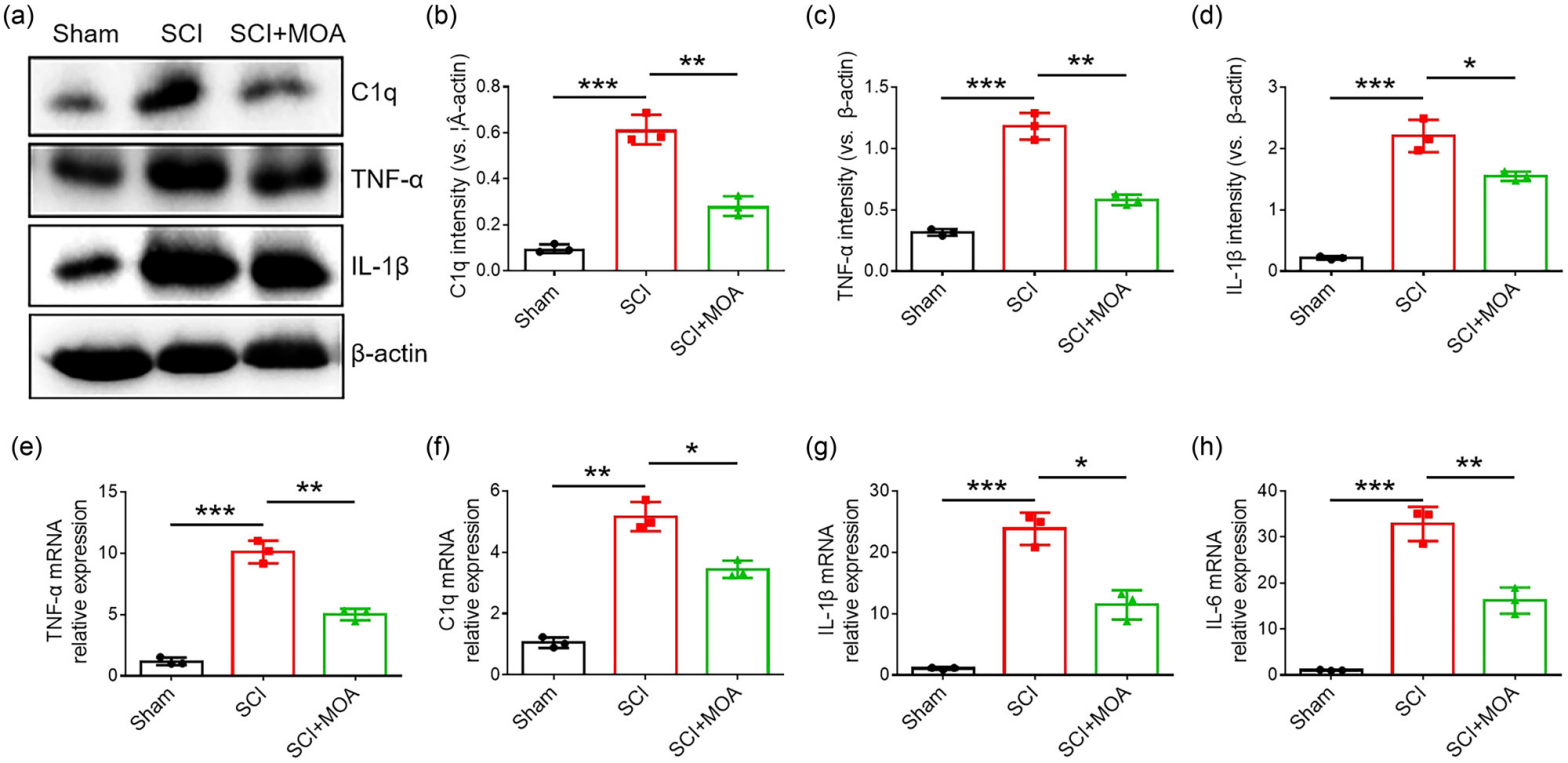
(a) Protein expression of C1q, TNF-α, and IL-1α in the spinal cords of rats in the three groups at 3 dpi. (b) The relative level of C1q protein expression in the three groups at 3 dpi, **p < 0.05. (c) The relative level of TNF-α protein expression in the three groups at 3 dpi, **p < 0.05. (d) The relative level of IL-1β protein expression in the three groups at 3 dpi, **p < 0.01. (e) TNF-α mRNA expression in the three groups at 3 dpi, **p < 0.05. (f) C1q mRNA expression in the three groups at 3 dpi, **p < 0.05. (g) IL-1β mRNA expression in the three groups at 3 dpi, **p < 0.05. (h) IL-6 mRNA expression in the three groups at 3 dpi, **p < 0.05.

Proinflammatory cytokines serve as important mediators of the pathogenesis of SCI. Accordingly, Western immunoblotting and qPCR were used to evaluate TNF-α, IL-1α, C1q, and IL-6 expression in these rats. All of these cytokines were upregulated in the spinal cord of SCI model rats, while they were effectively suppressed by MOA treatment (Figure 2).

### 3.4 MOA suppresses the maturation of M1 microglia in SCI model rats

**Figure 3:**
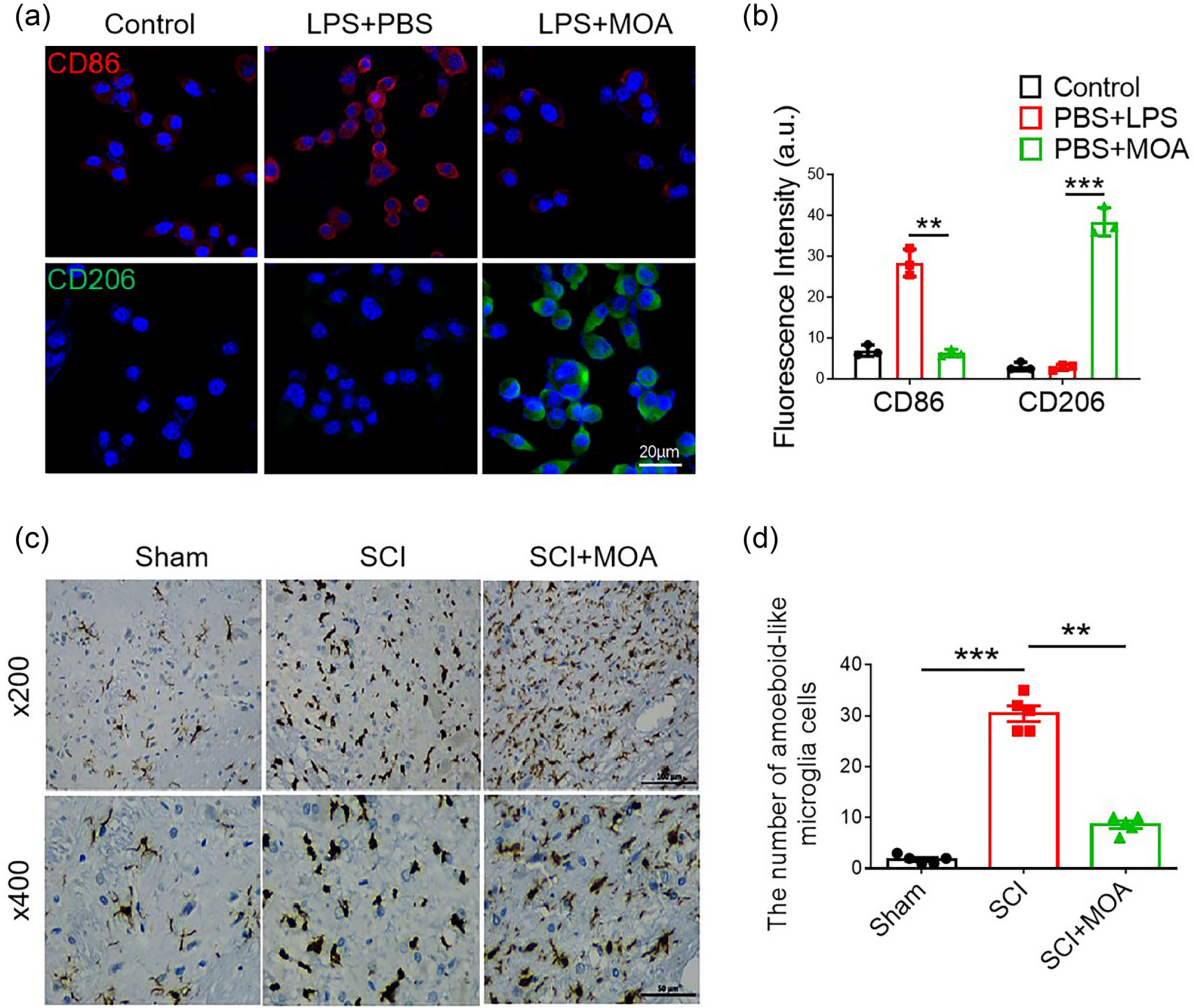
(a) Immunofluorescence-stained macrophages in the control, LPS + PBS, and LPS + MOA groups. M1 macrophage (CD86: red), M2 macrophage (CD206: green). (b) Fluorescence intensity of the control, LPS + PBS, and LPS + MOA groups, **, ***p < 0.05. (c) Immunohistochemical staining of Iba1 in Sham, SCI, and MOA groups 3 day following contusion. (d) The number of amoeboid-like microglia 3 day following contusion. **, ***p < 0.01, *n* = 5. Scale bar = 100 µm, scale bar = 50 µm.

The expression of CD86 was increased while that of CD206 was decreased significantly after treatment of macrophages with lipopolysaccharide (Figure 3a and b), MOA thus effectively regulated polarization of the macrophages. Under basal conditions, the microglia found within the spinal cord were ramified, exhibiting a characteristic branched morphology with small spherical cell bodies. After SCI, these native microglia were activated and differentiated into M1 microglia with amoeboid-like cell bodies with few or no ramified processes (Figure 3c). Relative to the SCI model group, significantly fewer amoeboid microglia were evident in the MOA-treated group, with M2-like microglia with more ramified processes instead evident in this group (Figure 3c and d). These results indicated the ability of MOA to suppress M1 maturation of microglia in the spinal cord.

### 3.5 MOA alleviates oxidative stress within the spinal cord following SCI

**Figure 4:**
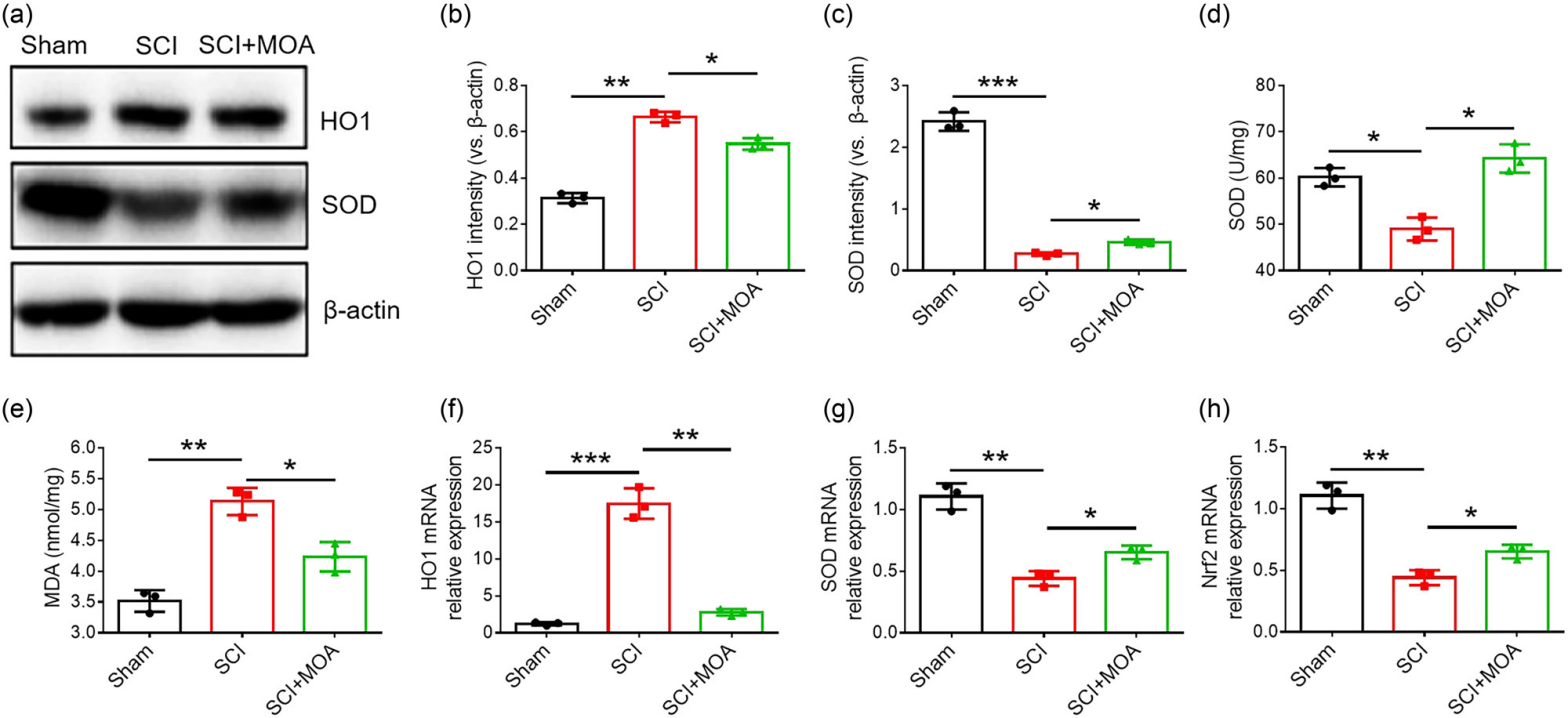
(a) Levels of HO-1 and SOD in the spinal cords of rats in the three groups at 3 dpi. (b) Relative levels of HO-1 level in the three groups at 3 dpi, *p < 0.01. (c) Relative levels of SOD expression in the three groups at 3 dpi, *p < 0.01. (d) SOD vigor level comparison at 3 day after contusion, *p < 0.05. (e) MDA vigor level comparison at 3 day after contusion, *p < 0.01 versus sham group and **p < 0.05 versus SCI group. (f) HO-1 mRNA expression in the three groups at 3 dpi, *p < 0.05. (g) SOD mRNA expression in the three groups at 3 dpi, *p < 0.05. (h) Nrf2 mRNA expression in the three groups at 3 dpi, *p < 0.05.

A significant increase in HO-1 levels was evident in the MOA group relative to the SCI group (*p* < 0.01) (Figure 4a and b), while SOD levels were reduced in the MOA group (*p* < 0.01) (Figure 4a and c). The respective SOD vigor levels in the sham, SCI, and MOA groups were 66.51 ± 3.68, 11.97 ± 2.49, and 53.42 ± 3.19 U/mg, with significant suppression of SOD activity in the SCI and MOA groups relative to the sham group (*p* < 0.01) but a significantly higher level of SOD activity in the MOA group relative to the SCI group (*p* < 0.01) (Figure 4d). Respective MDA levels in the sham, SCI, and MOA groups were 4.49 ± 0.21, 1.55 ± 0.29, and 3.58 ± 0.23 nmol/mg. These levels were highest in the SCI group, while in the MOA group, these levels were significantly below those in the SCI group but above those in the sham control group (*p* < 0.01) (Figure 4e). The mRNA expression of HO-1 was increased significantly in the SCI group and decreased significantly after MOA treatment (*p* < 0.05) (Figure 4f). The mRNA expression of SOD and Nrf2 was decreased significantly in the SCI group and increased significantly after MOA treatment (*p* < 0.05) ( Figure 4g and h).

### 3.6 MOA promotes the growth of neurons and inhibits the formation of glial scar

**Figure 5:**
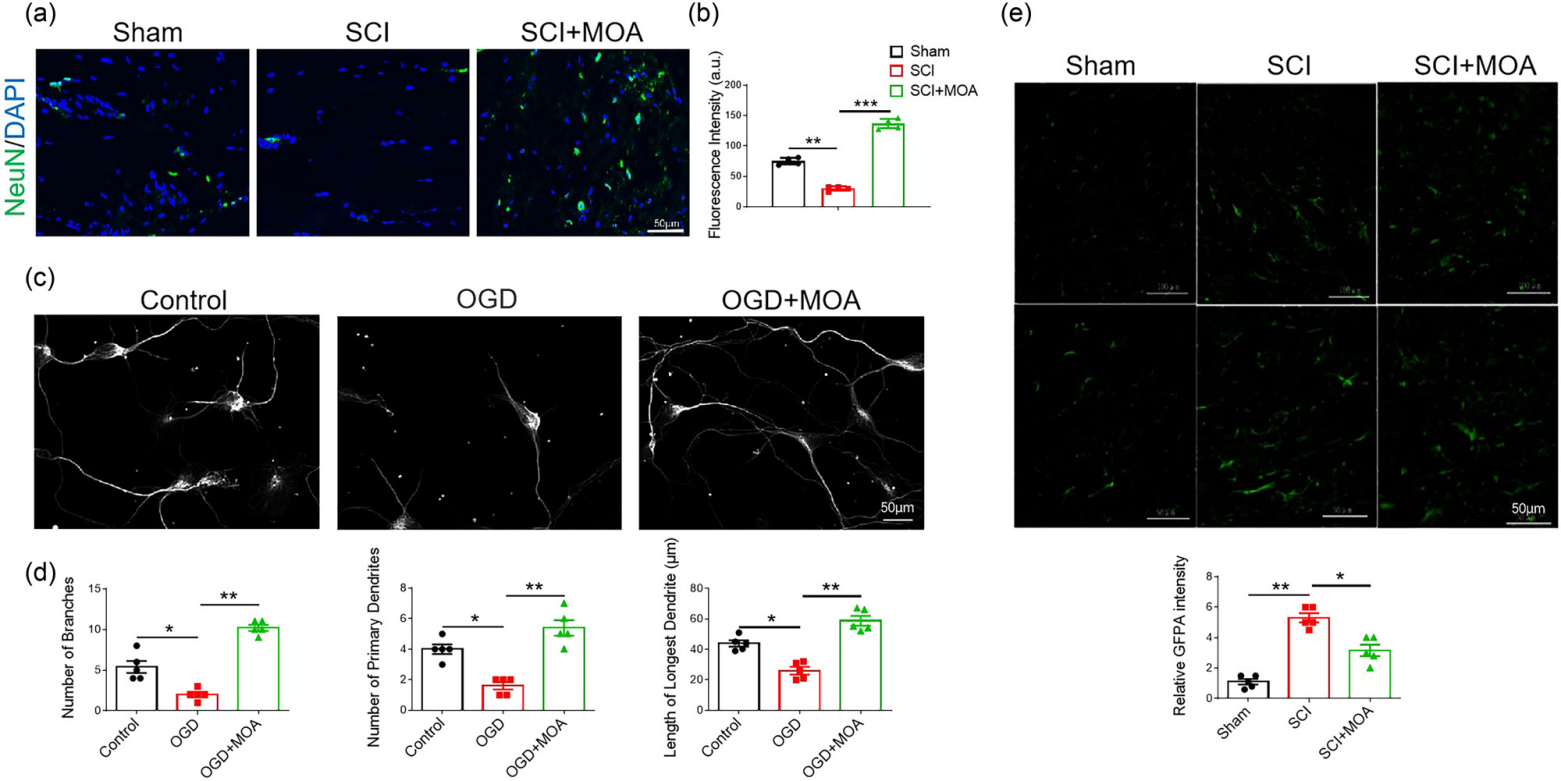
(a) Immunofluorescence-stained primary NSCs in the control, SCI, and SCI + MOA groups, Neurons (NeuN: green), Nuclei (DAPI: blue). (b) Fluorescence intensities in the control, SCI, and SCI + MOA groups, **p < 0.05, ***p < 0.05. (c) Neurons are labeled with anti-βIII-tubulin antibody for neuronal microtubules. (d) Numbers of branches in the control, OGD, and OGD + MOA groups, *p < 0.05, **p < 0.05. Numbers of primary dendrites in the control, OGD, and OGD + MOA groups, *p < 0.05, **p < 0.05. Lengths of longest dendrites in the control, OGD, and OGD + MOA groups, *p < 0.05, **p < 0.05. (e) Resting astrocytes in the Sham group, activated astrocytes in the SCI group at 3 d after contusion, and activated astrocytes in the MOA group at 21 d after contusion. Scale bar = 100 µm, scale bar = 50 µm. (f) Relative GFAP intensities in the three groups at 21 d after contusion, *p < 0.05, **p < 0.05.

Immunofluorescent staining showed that the number of neurons was significantly decreased after SCI but was increased significantly after MOA (Figure 5a and b). The numbers of branches and primary dendrites, as well as the length of the longest dendrites, were significantly reduced in neurons in the OGD group, while showing significant increases after MOA treatment (Figure 5c and d). At baseline, astrocytes in the spinal cord of rats in the sham control group exhibited small cell bodies and thin processes. Following SCI, these astrocytes were activated and exhibited pronounced morphological changes. Immunofluorescent staining indicated that astrocytes began undergoing polarization 3 days post-SCI, with accompanying cell body swelling and the thickening of cell processes. No significant morphological differences were observed between the SCI and MOA groups at 3 days post-injury. By day 21, there were significantly more astrocytes and significantly higher GFAP expression levels consistent with glial scar formation. Relative to the SCI group, astrocytes from MOA-treated rats exhibited larger cell bodies and elevated intracellular matrix levels (Figure 5e and f).

## 4 Discussion

Prior reports have found ozone to provide therapeutic benefits in the context of SCI, but the underlying molecular mechanisms have yet to be established [17]. While one previous study intraperitoneally injected rats with ozone [18], an MOA treatment strategy was instead employed in the present study given that MOA is widely used to administer ozone in clinical settings and is an established, safe procedure [19]. This study demonstrated that (1) MOA promoted the recovery of movement and bladder function, as well as the repair of local spinal cord tissue structure in the model group, (2) MOA alleviated oxidative stress resulting from SCI and reduced the expression of inflammatory factors such as TNF-α, IL-1β, and IL-6, as well as C1q, and (3) MOA inhibited the maturation of M1 microglia and the formation of glial scars while promoting the growth of neurons.

Relative to the SCI model group, rats that underwent MOA treatment exhibited significant improvements in BBB scores (*p* < 0.05), suggesting that MOA can promote motor recovery following SCI. Histological staining confirmed that while rats in the SCI group exhibited extensive spinal hemorrhage, edema, and cavity formation, these effects were significantly blunted in MOA-treated rats. MOA thus appears to protect against spinal tissue necrosis following traumatic SCI.

Christie et al. [20] found that acute oxidative stress can persist for 120 h following acute SCI in a rat model system. In clinical settings, methylprednisolone is prescribed within 24 h as a treatment for SCI, but other therapeutic options are vital to mitigate such oxidative stress after this 24 h post-injury interval has elapsed. Here, MOA treatment was found to enhance SOD activity while suppressing MDA levels. The transcription factor Nrf2 is associated with processes such as exogenous and endogenous metabolism, the inflammatory response, and oxidative stress. Huang et al. [21] found that up-regulation of the Nrf2/HO-1 pathway in diabetic rats could enhance antioxidant capacity. In this study, Nrf2 and HO-1expression was found to be significantly increased after MOA treatment in SCI rats, which indicated that MOA increased SOD activity and reduced the membrane lipid peroxidation possibly through up-regulating the expression of the Nrf2/HO-1 pathway. With the enhancement of the antioxidant capacity in the spinal cord, the secondary injury of spinal cord tissue was alleviated.

Following SCI incidence, microglia can rapidly proliferate and differentiate into neurotoxic M1 cells [22]. In the SCI model group, microglia presented with amoeboid morphology whereas in the MOA treatment group, these cells exhibited ramified processes. This suggests that the neuroprotective benefits of MOA treatment are associated with the M2 differentiation of microglia. M1 microglia can induce the development of A1 astrocytes by secreting proinflammatory factors including C1q, TNF-α, IL-6, and IL-1β, thereby modulating neuronal apoptotic activity [23]. In this study, CD86 levels were significantly increased in LPS-treated macrophages, with the macrophages exhibiting the M1 phenotype, while significant increases in CD206 accompanying the M2 phenotype were seen in the MOA-treated group. These results indicated that MOA could promote the differentiation of microglia into M2 phenotype, then the expression of TNF-α, IL-1, IL-6, and C1q were all significantly downregulated after the decline of M1 microglia differentiation.

In the study, the number of axons in the primary cultured neurons decreased significantly after OGD treatment, while axon numbers increased significantly in the MOA group. The number of neurons was also increased significantly in the MOA-treated group *in vivo*. These results indicate that MOA has a neuroprotective effect in SCI. Besides, reactive astrocyte polarization *in vivo* occurs 2–3 days following SCI, and the impact of MOA on astrocytes was thus analyzed in this experimental model system [24]. Analyses of astrocytes in the spinal tissue samples from these rats revealed that SCI models exhibited higher levels of GFPA expression and consistent astrocyte hypertrophy relative to the sham control group, in line with prior reports [25]. Reactive astrocytes in MOA-treated rats were more hypertrophic and exhibited a denser intracellular matrix as compared to those in the SCI model group. These changes may be associated with beneficial improvements in the astrocytes located proximal to the spinal lesion, given that MOA can improve the local oxygen supply and associated microcirculatory activity [26,27], further study still should be needed to reveal the mechanism.

At present, no regenerative therapies have been approved for the clinical treatment of SCI [28]. Hyperbaric oxygen therapy is generally initiated immediately following surgical decompression and methylprednisolone treatment in the clinic when managing SCI patients. However, some hospitals lack the necessary hyperbaric oxygen chambers, and other patients may be unable to undergo such treatment for a range of reasons. Ozone represents an easily obtainable and cost-effective alternative therapeutic tool that is not associated with any severe side effects such that it can readily be administered to patients. In this study, MOA effectively inhibited oxidative stress and inflammation. If further studies can validate the therapeutic benefits of MOA in an SCI patient cohort, then such treatment may help revolutionize the treatment of this extremely debilitating and intractable condition.

## 5 Conclusion

In summary, this study demonstrated that MOA has the potential to protect spinal cord integrity in the context of traumatic injury, potentially through anti-inflammatory, antioxidant, and regulatory effects that modulate the polarization of astrocytes and microglia. These beneficial effects indicate that MOA treatment represents an effective candidate for the clinical treatment of SCI.
